# Antinuclear Antibodies in Patients with Psoriatic Arthritis Treated or Not with Biologics

**DOI:** 10.1371/journal.pone.0134218

**Published:** 2015-07-31

**Authors:** Florent Silvy, Daniel Bertin, Nathalie Bardin, Isabelle Auger, Marie-Caroline Guzian, Jean-Pierre Mattei, Sandrine Guis, Jean Roudier, Nathalie Balandraud

**Affiliations:** 1 Rheumatology 1, IML, AP-HM, 270 Boulevard de Sainte Marguerite, 13009, Marseille, France; 2 Laboratoire d'immunologie, Pôle de Biologie, Hôpital de la Conception, Assistance Publique-Hôpitaux de Marseille, 147 boulevard Baille, 13005, Marseille, France; 3 INSERM UMRs 1076, UFR Pharmacie, Aix Marseille Université, 127 boulevard Jean Moulin, 13005, Marseille, France; 4 Laboratoire d’immunogénétique, INSERM UMRs 1097, Aix Marseille Université, 163 Avenue de Luminy, 13288, Marseille, France; Nippon Medical School Graduate School of Medicine, JAPAN

## Abstract

**Background:**

With the emergence of biotherapies, accurate diagnosis in early arthritis is needed. At this time, there is no biological marker of psoriatic arthritis.

**Objective:**

To test whether antinuclear antibodies (ANA) can be used as a diagnostic tool in psoriatic arthritis (PsA), we evaluated the prevalence of ANA in biologic-naïve PsA patients and in healthy blood donors.

**Methods:**

232 patients from the Rheumatology department, St Marguerite's Hospital, Marseilles, who fulfilled the CASPAR criteria for PsA, underwent clinical and laboratory investigations. Antinuclear antibodies (ANA), anti-extractable nuclear antigen antibodies (ENA), rheumatoid factor (RF), anti-citrullinated protein antibodies (ACPA) were assayed. Ninety-one healthy blood donors were also tested.

**Results:**

Detection of ANA by indirect immunofluorescence was significantly more frequent in sera from PsA patients than those from controls at serum dilution of 1:100 (57% compared with 40%, Odds Ratio (OR) 1.98 (1.2-3.4) p<0.02) and 1:160 (52% compared with 24%, OR 3,7 (1.9-7.2) p<0.001). No patients had lupus specific autoantibodies, 15 % had RF (34/232), and 1.7 % had ACPA (4/232).

**Conclusions:**

Detection of ANA was more frequent in sera from PsA patients than in those from healthy controls. This suggests that ANA could be a diagnosis orientation tool in PsA. Nevertheless, the specificity of these antibodies still remains to be investigated.

## Introduction

Psoriatic arthritis is inflammatory arthritis associated with psoriasis, a member of the spondylo-arthritis family [1]. Its diagnosis is not always obvious and relies on specific criteria, like CASPAR criteria [2–4]. The identification of biological markers of PsA would help its diagnosis.

Anti-citrullinated protein antibodies (ACPA) and antinuclear antibodies (ANA) have been reported in respectively 5% and 7–77% of patients with PsA [5–14]. Anti-TNFα agents, used for the treatment of psoriasis and PsA, may contribute to the development of ANA in these patients [15–24]

In this work, we studied: -1-The prevalence of ANA in 232 patients with PsA and 91 healthy controls and its evolution under anti-TNFα treatment. -2-The prevalence of anti-ENA, anti-dsDNA, RF and ACPA at baseline in PsA patients. -3-Whether ANA detection and clinical phenotype in PsA patients were correlated.

## Methods

### Patients

We retrospectively analyzed 232 patients followed at the Rheumatology department, St Marguerite's hospital, Marseille.

All consecutive venous blood samples (232 samples) from SpA patients, naïve of biologics and fulfilling the CASPAR criteria, sent to our university hospital immunology laboratory between November 2009 and March 2015 for routine detection of ANA were included in our study. After anti-TNF therapy, ANA were assayed once more in 34 patients.

Patients with any confounding disease, which could be associated with ANA positivity (for example: rheumatoid arthritis, systemic lupus erythematous, scleroderma, inflammatory bowel disease, dermatomyositis, auto immune thyroid disease, reactive arthritis) were excluded from this study.

### Clinical assessment

For every patient, we retrospectively collected clinical history including assessment of arthritis, concomitant illnesses and medications taken. Physical examination included a general medical examination with emphasis on skin, nails, peripheral and axial joints. The number of tender and swollen joints was recorded as well as the axial presence of syndesmophytes on X ray.

### Controls

Ninety-one healthy controls were recruited from the French National Blood Bank in Marseilles.

#### Ethics

Patients had routine tests. Serum samples and patient information were retrospectively analyzed. They were anonymized prior to analysis.

Healthy donors were registered at the French Blood Bank (ESF) under the number 20012-0098-7266. All participants gave written informed consent. Serum samples were anonymized prior to analysis. Sample collection and analysis (DC-2008-327) was approved by the « Cellule Bioéthique, direction générale pour la recherche et l’innovation, Ministère de l’Enseignement Supérieur et de la Recherche » (Ministry bioethics unit).

### Detection of anti nuclear antibodies and their specificity

Antinuclear antibodies (ANA) were detected by indirect immunofluorescence (IFI) on HEp-2 cells (Kallestad HEp-2 Cell Line Substrate, 12 well slides, Bio-Rad Laboratories, Hercules, CA) at a screening dilution of 1:100 for every patient and control. To be in compliance with the new recommendations regarding screening dilution, a sub group of patients and controls were further tested at a dilution of 1: 160 [25,26]. Since serum samples are only kept in our laboratory for one year, this analysis could only be done for 63 patients. We also analyzed 91 controls at this dilution.

Anti-ENA and anti-ds DNA antibodies were detected by commercially-available kits (EliA dsDNA; Phadia, Uppsala, Sweden; now part of Thermo Fisher Scientific).

RF was detected with the commercially available ELISA kit (ORGENTEC Diagnostika GmbH, Mainz, Germany).

In 34 patients under TNFα blockers, these tests were performed before and after anti-TNFα treatment.

### Statistical analysis

Comparisons between the 2 groups (PsA patients vs controls), with regards to demographic, clinical, biological and genetic factors, were carried out using chi-square tests incorporating Yates' correction for continuity when applicable. To evaluate the diagnostic value of ANA in psoriatic patients, data on sensitivity, specificity, positive predictive value and negative predictive value of the test were calculated. ROC curves could not be performed as IFI provided only qualitative data (positive or negative) in controls. R statistical software (R Foundation for Statistical Computing, Vienna, Austria) was used for data analysis. The statistical significance level was set at p = 0.05.

## Results

### Clinical features (Table 1)

**Table 1:** Clinical, biological, thoracolumbar X ray and genetic characteristics of 232 patients with PsA, serum tested for ANA at 1: 100.

Characteristics	Total n = 232	ANA + n = 132	ANA − n = 100	p value
**Gender**				
women	159 (69%)	99 (75%)	60 (60%)	**p<0.02**
men	73 (31%)	33 (25%)	40 (40%)	NS
**Age at blood sample** (average, min-max)	46 (18–70)	46 (18–78)	47 (18–70)	NS
**Age at onset PsA** (average, min-max)	40 (18–65)	41 (16–65)	40 (15–65)	NS
**Duration of PsA** (average, min-max)	5 (0–37)	5 (0–24)	6 (0–37)	NS
**Psoriasis** (personal history) n (%)	194 (83%)	110 (83%)	84 (84%)	NS
**Arthritis pattern at assessment**, n (%)				
dactylitis	56 (24%)	28 (21%)	28 (28%)	NS
peripheral	71 (30%)	46 (34%)	25 (25%)	**p<0.2**
axial only	21 (9%)	13 (6%)	8 (4%)	NS
axial and peripheral	127 (55%)	68 (30%)	60 (26%)	NS
unknown	13 (6%)	5 (2%)	8 (3%)	NS
**Axial syndesmophytes on thoracolumbar X ray**	23 (10%)	13 (10%)	10 (10%)	NS
**Biological features** (%)				
**RF +**	34 (15%)	23 (17%)	11 (11%)	NS
**Anti Ds DNA +**	0	0	0	NS
**Anti SSA**	3 (0.5%)	3 (0.5%)	0	NS
**Anti SSB**	0	0	0	NS
**Anti RNP**	0	0	0	NS
**Anti CCP +** (>50UI/L)	4 (1.7%)	2 (0.5%)	2 (0.5%)	NS
**HLA-B27**	16%	16,80%	15%	NS
**HLA-DR Shared Epitope**	36%	35%	38%	NS
**HLA-DR7**	31%	34%	27%	NS
**HLA-CW6**	29%	31%	26%	NS

The PsA group was comprised of 232 patients, 159 women and 73 men, with a mean age of 46 years old (median: 47 years old, range: 18–70 years old) and the control group was comprised of 91 healthy blood donors, with a mean age of 43 years old (median: 43 years old, range: 21–69 years old), sex ratio 1.5. Pure peripheral joint involvement was seen in 30%, pure axial involvement in 9% and involvement of both in 55% of patients (data unknown for 6%).

Twenty-four percent of patients had at least one dactylitis, 83% had skin or nail psoriasis.

Family history of psoriasis was present in 38.5% of patients. Details available on S1 Table.

### Axial radiological features

Non-marginal syndesmophytes on the thoracolumbar spine were detected on the X rays of 10% of patients.

### ANA Detection

Prior to any biologics, serum from 132 of 232 (57%) PsA patients and 28 of 70 (40%) healthy controls was positive for ANA at dilution 1:100 (OR 1.98 (1.2–3.4) p<0.02). Titers ranged from 1:100 to 1:800 (Table 2). To evaluate the predictive value of ANA at 1:100, we calculated sensitivity at 58%, specificity at 64%, positive predictive value at 62% and negative predictive value at 60%

**Table 2:** Frequency of ANA in serum from PsA patients and healthy controls at dilutions 1: 100.

Biological feature	PsA pos 1: 100	Controls pos 1:100	OR	CI at 95%	**p value**
**Number tested**	**n = 232**	**n = 70**			
**Total**	132/232 (57%)	28/70 (40%)	2.0	1.2–3.4	p<0.02
**Women**	99/159 (62%)	15/28 (53%)	1.4	0.6–3.2	NS
**Men**	33/73 (45%)	12/40 (30%)	1.9	0.8–4.0	NS

Prior to any biologics, 33 of 63 (52%) PsA patients and 21 of 91 (23%) healthy controls were positive for ANA at dilution 1:160 (OR 3.7 (1.9–7.2) p<0.001) (Table 3). To evaluate the predictive value of ANA at 1:160, we calculated sensitivity at 62%, specificity at 71%, positive predictive value at 54% and negative predictive value at 77%.

**Table 3:** Frequency of ANA in serum from PsA patients and healthy controls at dilutions 1: 160.

Biological feature	PsA pos 1: 160	Controls pos 1:160	OR	CI at 95%	p value
**Number tested**	**n = 63**	**n = 91**			
**Total**	33/63 (52%)	21/91 (23%)	3.7	1.9–7.2	p<0.001
**Women**	26/46 (57%)	8/34 (23%)	4.2	1.6–11.2	p<0.01
**Men**	8/17 (47%)	12/55 (22%)	3.2	1.0–10.0	p<0.05

For PsA patients ANA were more frequently detected in women than in men: 62% in women and 45% in men at 1:100, 57% in women and 47% in men at 1:160.

In 95% of patients, ANA displayed a constant speckled pattern. In 5% of patients, ANA also had a nucleolar speckled pattern.

None of the patients developed either clinical symptoms of systemic lupus erythematous or biological signs (absence of anti-dsDNA, antiSm, anti-SSB or anti-RNP antibodies). Among 232 patients, only 3 patients were positive for anti-SSA/Ro antibodies. Rheumatoid factor was found in 34 patients and anti-CCP antibodies in 4 patients with titers above 50 IU/mL (normal <20 IU).

### HLA genetic background

The frequencies of HLA-B27, HLA-DR7, HLA-Cw6, the HLA-DRB1 shared epitope were not significantly different in PsA patients with regards to ANA status.

### ANA follow-up under anti-TNF treatment in PsA patients

We followed 34 PsA patients under treatment with anti-TNF. Before treatment, 20 were ANA negative and 14 were ANA positive.

After a mean of 58 months (median: 32 months) of treatment with anti-TNF, 11/ 20 (55%) of ANA negative patients became positive. Among the 14 patients who had ANA before anti-TNF treatment, all remained positive and 5 increased their ANA titer (from 5 first detected at 1:100, 2 became positive at 1:200, 2 at 1:400, and 1 at 1:800)

## Discussion

Psoriatic arthritis, a condition defined by the association of psoriasis and inflammatory arthritis, suffers from the lack of any unambiguous diagnostic marker. This is especially important nowadays as the range of treatments for arthritis has been significantly improved by biologics, the usage of which is usually restricted to well characterized types of arthritis: rheumatoid arthritis, ankylosing spondylitis, psoriatic arthritis.

Here, we wondered whether antinuclear antibodies could be of some help in diagnosing psoriatic arthritis.

We found that, if one sets positivity at a titer of 1:160, more than half of the patients with psoriatic arthritis and less than a quarter of healthy controls have ANA in their sera.

These findings are not entirely new: indeed, as early as 1966, Sonnischen reported that 77% of patients with psoriatic arthritis had ANA [27] even if these first results, given at a dilution of 1:40 could not be reproduced. In other series, patients were mostly screened at a dilution of 1:80 [28]. Other groups which have tested for ANA in patients with psoriasis (with or without arthritis), at a screening dilution of 1:80, reported respectively that 11%, 34%, 9%, 22% of patients had ANA at baseline [17,20,22–24]

However, our study is the first providing a healthy control group and a patient group tested at the same time. Previous studies just compared their findings with epidemiologic data obtained from the general population. The frequency of ANA was higher in women than in men in both the PsA and the healthy control groups, as previously described [29].

ANA tended to be associated with peripheral disease even if this feature did not reach significance.

In 34 patients tested before and after biotherapy, 11/20 (55%) negative sera before anti-TNF treatment became ANA positive after treatment. This is in accordance with previous studies: in the University of Toronto’s prospective PsA cohort autoantibodies developed in 8/16 PsA patients treated with infliximab [15]. In psoriatic patients treated with infliximab (with or without arthritis), the prevalence of ANA always increased: respectively from 11 to 72% [17], from 9 to 48% [30], and from 22 to 63% [22] with a gradual increase both in ANA titer and in percentage of ANA pattern even restricted for anti dsDNA and anti nucleosome [20,22]. Interestingly, in the study by Lora et al., patients who developed ANA positivity showed a faster clinical response to infliximab. Contrary to these findings, Hoffmann et al. showed that higher pretreatment ANA titers were associated with a loss of response to infliximab and induction of anti-infliximab antibodies. In our study, we could not find any correlation between ANA positivity and clinical evolution.

## Conclusion

More than half of the patients and less than 25% of healthy controls tested positive for ANA at a serum dilution of 1:160 in a series of 232 patients with PsA and 91 healthy controls. Further studies are needed to characterize ANA fine specificity and turn them into a diagnostic tool.

## Supporting Information

S1 Table Clinical and biological data of PsA patients. (PDF)
