# Supplementary material for: Antinuclear Antibodies in Patients with Psoriatic Arthritis Treated or Not with Biologics
Source: PLoS One. 2015 Jul 31;10(7):e0134218. doi: 10.1371/journal.pone.0134218 (PMC4521886; doi:10.1371/journal.pone.0134218)
Supplement: S1 Table — (PDF) [file pone.0134218.s001.pdf]

tient Numb	Sexe	HLA-B	HLA-DR	HLA -C	ANA POS 1: 100	ANA POS 1: 160	Age at onset
1	F	38/53	102/13	4/12	1:800	1:1280	NR
2	F	35 /-	NT	NT	1:800	ND	65
3	F	51/ 51	7/12	15/ 15	1:800	ND	14
4	F	8/ 37	301/1454	6 /7	1:800	ND	18
5	F	8/ 52	03/14	7/ 7	1:800	ND	43
6	F	27/63	401/1103	1/ 3	1:800	ND	45
7	H	NT	NT	NT	1:800	1:640	40
8	F	35/49	01/1302	4/ 7	1:400	1:640	NR
9	F	39/ 55	11/16	3/ 12	1:400	1:320	NR
10	H	27 /60	01/ 0401	NT	1:400	ND	30
11	F	18/21	301/1001	5/ 7	1:400	ND	60
12	F	8/ 27	3/ 7	6 / 7	1:400	ND	46
13	F	7/ 35	13/15	4 / 7	1:400	ND	45
14	F	35/53	13/15	4/NT	1:400	ND	45
15	f	44/44	0701/1101	16/16	1:400	ND	NR
16	F	15/51	13/13	7/16	1:400	ND	50
17	F	27/50	07/11	NT	1:400	ND	42
18	F	14/18	102/102	8/15	1:400	ND	26
19	F	40/44	701/1302	NT	1:400	ND	54
20	F	39/ 50	102/07	6 12	1:400	ND	13
21	F	27/49	404/07	2/ 7	1:400	ND	51
22	F	7/ 40	1101/1501	2/ 7	1:400	ND	50
23	F	7/58	101/101	15/7	1:400	ND	45
24	F	8/27	11/14	2/ 7	1:400	ND	40
25	F	15/ 27	101/701	1 / 14	1:400	ND	30
26	H	40/35	07/13	2/ 4	1:400	ND	30
27	H	7/ 40	0101/0401	7/15	1:400	ND	52
28	H	7/ 27	701/1501	1 /7	1:400	1:320	54
29	F	07/35	01/13	NT	1:200	1:320	55
30	F	07/13	07/15	6/7	1:200	1:320	35
31	F	7/35	103/1501	4/ 7	1:200	1:320	NR
32	F	08/27	01/15	1/7	1:200	1:320	NR
33	F	8/ 35	03/11	4/ 7	1:200	1:320	NR
34	F	8/18	01/03	7/NT	1:200	1:320	30
35	F	14/45	01 11	8 /16	1:200	1:320	NR
36	F	39/49	07/1601	7/ 7	1:200	1:320	45
37	F	7/14	07/08	7/8	1:200	1:320	NR
38	H	27/NT	11/12	2/8	1:200	1:320	NR
39	H	27/NT	01/11	NT	1:200	1:160	NR
40	H	38 /51	NT	12/14	1:200	1:320	NR
41	H	14/45	0405/701	6/8	1:200	1:320	NR
42	H	18 /51	03/11	5/15	1:200	ND	NR
43	F	7/ 27	1301/1501	NT	1:200	ND	52
44	F	08/51	11/15	7/15	1:200	ND	41
45	F	45/49	405/1101	6/ 7	1:200	ND	NR
46	F	NT	NT	NT	1:200	ND	36
47	F	13/ 44	1101/1502	5/6	1:200	ND	60
48	F	44/53	402/03	NT	1:200	ND	50
49	F	50/58	405/13	7/14	1:200	ND	35

50	F	7/ 8	15/15	7/ 7	1:200	ND	47
51	F	50/ 51	13/15	6/16	1:200	ND	45
52	F	NT	NT	NT	1:200	ND	28
53	F	18/50	07/14	NT	1:200	ND	45
54	F	7/ 27	1001/1501	6/12	1:200	ND	50
55	F	40/ 41	13/16	3 /17	1:200	ND	38
56	f	35/35	0301/1401	4/ 4	1:200	ND	18
57	F	40/44	102/08	2/ 3	1:200	ND	45
58	F	35/44	07/11	NT	1:200	ND	30
59	F	13/44	07/14	6/ 5	1:200	ND	48
60	F	27/45	0701/1501	2/ 6	1:200	ND	45
61	F	49/ 50	7/11	6/ 7	1:200	ND	62
62	H	8/35	03/03	NT	1:200	1:320	30
63	F	35/35	1401/1401	NT	1:100	1:160	NR
64	F	27/35	15/16	2/ 4	1:100	ND	NR
65	F	14/49	102/13	7/8	1:100	1:160	28
66	F	39/55	NT	3/12	1:100	1:160	NR
67	F	14/45	0405/701	6/ 8	1:100	1:160	NR
68	F	18/ 57	10/11	6/NT	1:100	1:160	NR
69	F	8/35	03/04	7/12	1:100	1:160	30
70	F	NT	4 /13	NT	1:100	1:160	15
71	F	18/44	7/11	NT	1:100	1:160	30
72	F	8/48	NT	NT	1:100	1:160	50
73	F	27/50	1/ 7	6/NT	1:100	1:160	63
74	F	15/53	13/15	4/ 7	1:100	ND	65
75	F	58/58	13/13	6/3	1:100	ND	45
76	F	37/39	403/11	6/12	1:100	ND	55
77	H	7/38	14/15	NT	1:100	1:160	NR
78	H	07/40	08/15	3/ 7	1:100	ND	55
79	H	NT	NT	NT	1:100	ND	55
80	F	18/ 44	0101/301	5/5	1:100	ND	28
81	F	13/38	1401/1501	6/12	1:100	ND	42
82	F	13/51	07/11	6/16	1:100	ND	30
83	F	38/57	0401-07	NT	1:100	1:160	52
84	F	18/27	03/16	2/ 5	1:100	ND	20
85	F	13/49	101/701	6/ 6	1:100	ND	10
86	F	13/ 57	7/ 7	6/6	1:100	ND	50
87	F	51/57	7/14	NT	1:100	ND	45
88	F	49/53	13/13	4/ 7	1:100	ND	30
89	F	35/NT	407/1454	NT	1:100	ND	28
90	F	27/39	101/11	NT	1:100	ND	40
91	F	27/55	3/16	2/3	1:100	ND	55
92	F	13/44	7/13	3/6	1:100	ND	35
93	F	18/50	701/1301	6/14	1:100	ND	45
94	F	44/ 53	1201/1303	4/ 5	1:100	ND	52
95	F	35/40	404/13	6/NT	1:100	ND	NR
96	F	18/44	0102/11	NT	1:100	ND	20
97	F	8/49	03/11	NT	1:100	ND	55
98	F	8/ 55	07/11	1/ 7	1:100	ND	30

99	F	14/ 51	07/13	NT	1:100	ND	33
100	F	7/53	14/15	4/ 7	1:100	ND	55
101	F	8/ 70	11/15	2/ 7	1:100	ND	43
102	F	14/ 35	102/1301	NT	1:100	ND	50
103	F	15/ 44	701/701	12/16	1:100	ND	35
104	F	35/ 44	701/1104	4 /4	1:100	ND	25
105	F	27/ 52	12/15	3 /12	1:100	ND	33
106	F	14/ 18	102/1501	8 /12	1:100	ND	35
107	F	13/ 18	15/15	4/12	1:100	ND	54
108	F	39/62	13/03	NT	1:100	ND	34
109	F	14/ 62	101/701	4 /8	1:100	ND	37
110	F	44/ 55	7/12	1/ 16	1:100	ND	50
111	F	27/-	NT	NT	1:100	1:160	34
112	F	38/39	1301/1601	12/12	1:100	ND	20
113	F	NT	NT	NT	1:100	ND	30
114	H	NT	NT	NT	1:100	ND	50
115	H	45/ 50	402/7	6/ 5	1:100	1:160	60
116	H	53/35	0101/13	NT	1:100	ND	52
117	H	44/ 51	13/13	NT	1:100	1:160	25
118	H	45/49	0401/11	6/ 7	1:100	ND	30
119	H	38/ 40	404/1101	3 /12	1:100	ND	48
120	H	35/50	701/1101	NT	1:100	ND	33
121	H	08/14	101/0301	7/ 8	1:100	ND	55
122	H	37/52	401/08	NT	1:100	ND	48
123	H	15/ 51	401/1104	3/ 16	1:100	ND	60
124	H	7/ 40	301/301	3 /7	1:100	ND	50
125	H	50/57	07/NT	6/NT	1:100	ND	20
126	H	8/ 13	701/1301	6/7	1:100	ND	25
127	H	13/ 52	403/7	6 /12	1:100	ND	40
128	H	35/58	1302/1401	4/ 7	1:100	ND	50
129	H	8/ 44	3/7	4 7	1:100	1:160	40
130	H	18/44	07/11	NT	1:100	ND	25
131	H	2/ 18	0403/14	NT	1:100	ND	28
132	H	35/51	1 11/14	NT	1:100	ND	40
133	F	50/58	405/13	NT		ND	35
134	F	8/60	13/03	3/ 7		ND	46
135	F	58/ 52	1/15	NT		0	25
136	F	18/39	13/16	7/12		ND	40
137	F	44/49	405/11	5/ 7		0	35
138	F	51 72	03/04:02	6/15		ND	NR
139	F	46569	8/11	15/16		ND	54
140	F	08/40	01, 15	NT		0	40
141	F	47/67	101/113	NT		0	NR
142	F	44/51	7/13	NT		ND	51
143	F	15/ 40	404/701	3 /4		0	30
144	F	7/ 7	NT	NT		0	NR
145	F	35/55	01/14	1/4		ND	NR
146	F	44/57	401/701	NT		0	35
147	F	13 49	07/11	6 /7		0	NR

148	F	18/39	01/11	7/12		ND	33
149	F	27/ 27	08/15	2/ 2		ND	14
150	F	7/14	102/11	NT		ND	37
151	F	35/35	101/1401	NT		0	50
152	F	39/51	08/09	NT		ND	NR
153	F	27/38	11/ 13	NT		ND	45
154	F	08/15	103/111	NT		ND	30
155	F	8/57	7/15	6/ 7		ND	35
156	F	61/63	1302/1601	7/ 7		ND	50
157	F	15/22	1 4/11	1/ 3		ND	65
158	F	37/55	301/1101	6 3		ND	25
159	F	15/56	11/13	1/ 2		0	44
160	F	15/44	07/12	3/ 5		ND	30
161	F	8/13	301/1104	6/ 7		0	45
162	F	NT	NT	NT		ND	55
163	F	8/ 49	1501/1501	7 /7		ND	40
164	F	NT	102/11	7 /7		ND	55
165	F	14/35	102/14	4/8		0	30
166	F	7/50	103/108	6/15		ND	36
167	F	41/51	03/04:05	6/15		ND	30
168	F	45/57	7/13	NT		0	50
169	F	7/ 35	14/16	4/ 7		ND	45
170	F	38/57	07/13	6/12		ND	40
171	F	18/18	101/103	5/ 7		ND	47
172	F	18/4	11/14	5/15		ND	40
173	F	NT	NT	NT		ND	55
174	F	8/18	0101/1501	7/ 8		ND	35
175	F	22/ 51	1001/ 11	6neg		ND	53
176	F	7/ 7	1301/1501	7/ 7		ND	55
177	F	18/44	07/16	6 /4		ND	16
178	F	7/ 27	801/1101	15/ 16		ND	50
179	F	7/ 38	1301/1501	NT		ND	55
180	F	8/62	401/03	NT		0	50
181	F	07/38	01/07	6/ 7		ND	21
182	F	NT	NT	NT		ND	42
183	F	18/44	07/11	NT		0	47
184	F	44/49	07/11	7/16		ND	40
185	F	NT	301/1501	NT		ND	46
186	F	51/51	08/08	14/15		ND	40
187	F	8/58	11/13	7/7		ND	57
188	F	44/51	7/15	NT		0	30
189	F	50/51	0403/1201	6/14		ND	50
190	F	14/35	07/14	NT		0	40
191	F	7/ 37	701/1302	6/7		0	20
192	F	27/ 60	103/11	1/ 3		ND	25
193	H	08/35	03/07	4/ 7		0	NR
194	H	18/ 35	101/1104	NT		ND	31
195	H	44/NT	NT	NT		ND	49
196	H	18/18	101/101	5/ 7		0	44

197	H	27/57	01/07	2/ 6		0	35
198	H	NT	NT	NT		ND	40
199	H	8/ 14	7/14	7 /8		ND	52
200	H	49/ 50	806/1104	NT		ND	50
201	H	NT	NT	NT		ND	40
202	H	18/ 57	NT	NT		ND	42
203	H	49/50	7/13	4/7		ND	00
204	H	39/41	11/13	7/17		0	45
205	H	44/51	07/07	4/15		ND	45
206	H	39/39	101/0401	8/12		0	54
207	H	35/ 51	1501/1502	4/ 14		ND	45
208	H	27/35	101/1101	1/ 7		ND	45
209	H	8/ 27	03/03	2 / 7		ND	26
210	H	14/15	101/102	3/ 3		ND	28
211	H	27/40	401/1101	2/ 2		ND	20
212	H	35/50	04/04	NT		ND	55
213	H	27/51	NT	2/14		ND	17
214	H	27/51	07/08	NT		ND	34
215	H	27/44	301/1404	2/ 2		ND	35
216	H	7/ 35	1101/1103	4 /7		ND	41
217	H	7/44	103/1104	4/ 7		ND	50
218	H	NT	NT	NT		ND	45
219	H	7/44	1101/1101	7/7		ND	42
220	H	07/14	0101/14	7/ 8		ND	45
221	H	18/ 27	301/1101	NT		ND	33
222	H	8/ 39	101/301	NT		ND	53
223	H	35/63	NT	NT		ND	43
224	H	51/57	07/10	6/ 1		0	70
225	H	7/ 55	13/15	NT		ND	19
226	H	14/51	0102/11	NT		ND	47
227	H	8/ 37	801/1001	6/ 7		ND	40
228	H	27/35	101/101	NT		ND	55
229	H	27/ 51	NT	NT		ND	40
230	H	57/60	07/11	NT		ND	28
231	H	8/44	03/11	7/7		ND	35
232	H	37/57	0101/07	6/ 6		ND	45

duration (years)	ANTI ENA	ACPA	RF	Dactilytis	Enthesitis	familial psoriasis	psoriasis
NR	neg	nef	37	0	hill	no	hand foot
6	neg	neg	neg	1	no	no	leg
0	neg	neg	neg	1	gluteus	yes	no
1	neg	neg	neg	0	gluteus	yes	skin
11	neg	neg	neg	0	yes	no	hand
0	neg	neg	neg	0	elbow	no	hair
1	neg	neg	neg	NR	no	no	skin
NR	neg	49	neg	NR	hill	no	no
NR	neg	neg	27	0	no	no	diffuse
4	neg	neg	neg	NR	no	no	no
6	neg	neg	neg	0	hill	no	nail
0	neg	neg	50	0	no	no	no
1	neg	neg	78	0	no	no	no
4	neg	neg	neg	0	hill	yes	no
NR	neg	neg	neg	0	hill	no	hair
4	neg	neg	neg	0	gluteus	no	nail
1	neg	nég	nég	0	no	no	hair, foot
1	neg	neg	neg	0	no	yes	hand foot
4	neg	neg	neg	1	no	yes	hair
0	neg	neg	neg	0	hill	yes	no
0	neg	neg	neg	1	no	no	elbow,
3	SSA pos 16	neg	neg	0	gluteus	no	diffuse
4	neg	neg	neg	0	no	no	no
20	neg	neg	50	0	no	no	diffuse
5	neg	neg	neg	0	no	no	diffuse
7	neg	neg	neg	1	no	no	diffuse
11	neg	neg	neg	0	no	yes	leg
5	neg	neg	neg	0	no	yes	diffuse
3	neg	neg	27	0	no	no	nail
2	neg	neg	neg	0	no	no	diffuse hair
NR	neg	neg	neg	1	no	no	diffuse
NR	SSA pos	neg	34	0	no	yes	diffuse
NR	ssa pos	neg	neg	0	no	yes	nail ?
7	neg	neg	neg	0	no	yes	diffuse
NR	neg	neg	20	1	no	no	diffuse
0	neg	neg	neg	0	no	no	diffuse hair
NR	neg	neg	23	1	no	no	diffuse
NR	neg	neg	74	1	hill	yes	diffuse
NR	neg	neg	neg	1	no	no	diffuse
NR	neg	neg	neg	0	multiple	no	cc elbow
NR	neg	neg	neg	0	multiple	yes	diffuse
NR	neg	neg	35	0	no	yes	cc elbow
2	neg	neg	pos 29	0	no	yes	diffuse
0	neg	neg	31	0	hill	no	ear
NR	neg	neg	neg	0	hill	no	diffuse
0	neg	neg	neg	0	hill	yes	diffuse nail
16	neg	neg	neg	1	gluteus	no	hair
2	neg	neg	neg	0	?	no	foot
5	neg	neg	neg	0	gluteus	yes	diffuse

1	neg	neg	neg	0	hill	yes	diffuse
2	neg	neg	neg	0	hill	?	hair
1	neg	neg	neg	1	hill	yes	diffuse
3	neg	neg	neg	NR	no	no	no
7	neg	neg	neg	1	hill	yes	no
1	neg	neg	neg	0	no	no	no
45	neg	neg	neg	1	no	no	no
5	neg	neg	neg	0	no	no	diffuse
5	neg	neg	neg	0	no	yes	diffuse
8	neg	neg	neg	0	no	no	diffuse
2	neg	neg	neg	0	hill	no	hair
17	neg	neg	neg	0	no	no	no
1	neg	neg	neg	0	no	no	diffuse
NR	neg	neg	neg	0	hill	no	hair nail
NR	SSA pos 38	neg	neg	0	hill	no	diffuse
10	neg	neg	neg	1	gluteus	no	nail
NR	neg	neg	25	0	no	no	diffuse hand
NR	neg	neg	neg	0	no	yes	no
NR	neg	neg	pos 21	0	no	no	hair
6	neg	neg	neg	0	no	no	hair
0	neg	neg	neg	0	no	no	no
16	neg	neg	neg	0	no	no	diffuse
0	neg	neg	neg	1	no	no	hair elbow knees
5	neg	neg	neg	1	gluteus	no	diffuse
3	neg	neg	neg	0	no	yes	diffuse+++
9	neg	neg	neg	0	no	no	diffuse
9	neg	neg	neg	0	no	yes	diffuse nail
NR	neg	27	neg	0	no	no	elbow
15	neg	neg	25	0	no	no	diffuse
5	neg	neg	36	NR	no	no	no
1	neg	neg	neg	0	no	yes	diffuse
2	neg	neg	20	0	no	yes	no
0	neg	neg	neg	1	multiple	yes	diffuse
4	neg	neg	neg	0	no	no	knee elbow
13	neg	neg	neg	0	gluteus	no	leg
7	neg	neg	neg	0	no	no	no
18	neg	neg	neg	0	multiple	yes	diffuse
2	neg	neg	neg	1	multiple	no	hair elbow diffuse
7	neg	neg	neg	1	multiple	no	hair,hand
3	neg	neg	neg	0	hill	yes	diffuse
11	neg	neg	neg	0	no	yes	nail
6	neg	neg	pos 30	1	wrist	?	diffuse
6	neg	neg	neg	0	hill	?	diffuse
8	neg	neg	neg	0	hill	yes	diffuse
2	neg	neg	50	0	no	no	elbow hair
NR	neg	neg	neg	0	no	no	diffuse
0	neg	neg	neg	1	no	no	no
5	neg	neg	neg	0	no	no	hair
3	neg	neg	neg	0	multiple	no	foot

2	neg	neg	neg	0	no	no	hair
5	neg	neg	neg	0	no	yes	diffuse
7	neg	neg	neg	0	hill	?	knee nail
5	neg	neg	neg	0	no	no	diffuse
4	neg	neg	neg	0	no	yes	hair
1	neg	neg	neg	1	hill	no	diffuse
3	neg	neg	neg	0	hill	no	hair
2	neg	neg	neg	0	hill	no	diffuse
0	neg	neg	neg	0	no	no	no
7	neg	neg	neg	0	hill	yes	diffuse
0	neg	neg	neg	NR	no	no	no
2	neg	neg	neg	0	no	no	diffuse
0	neg	neg	neg	0	hill	no	diffuse
1	neg	neg	neg	0	hill	no	diffuse
2	neg	neg	neg	0		yes	diffuse elbow
9	neg	neg	neg	0	hill	no	diffuse nail hair
0	neg	neg	24	0	?	no	hair
10	neg	neg	22	1	gluteus	no	diffuse hand
2	neg	neg	neg	0	wrist	yes	diffuse
5	neg	neg	neg	0	no	no	diffuse hair nail
0	neg	neg	neg	0	no	no	diffuse
0	neg	neg	neg	0	gluteus	yes	diffuse
1	neg	neg	neg	1	multiple	no	hand
0	neg	neg	neg	0	hill	no	hair
6	neg	neg	neg	0	no	?	diffuse
12	neg	neg	neg	0	0	no	diffuse
1	neg	neg	neg	0	gluteus	yes	diffuse
37	neg	neg	neg	0	no	?	diffuse
4	neg	neg	neg	0	no	?	?
1	neg	neg	24	1	no	?	hand foot
0	neg	neg	neg	0	no	?	naill hand foot
15	neg	neg	neg	1++	knee	yes	diffuse
19	neg	neg	neg	0	wrist	no	knee
4	neg	neg	neg	0	no	?	knee
24	neg	neg	neg	0	no	no	
0	neg	neg	neg	0	no	no	nail hand foot
0	neg	neg	neg	0	hill	yes	no
7	neg	neg	neg	0	hill	?	diffuse
1	neg	neg	neg	1	hill	yes	diffuse
NR	neg	neg	neg	1	no	no	diffuse
2	neg	neg	34	0	no	no	diffuse
6	neg	neg	neg	0	no	no	no
NR	neg	neg	neg	0	no	yes	no
0	neg	neg	neg	0	multiple	no	hair
5	neg	neg	neg	0	gluteus	yes	diffuse
	neg	neg	neg	0	multiple	yes	hair
6	neg	neg	neg	1	multiple	yes	diffuse
0	neg	neg	neg	1	no	no	hair
5	neg	neg	neg	0	gluteus	yes	elbow

9	neg	neg	neg	1	no	yes	hair nail
4	neg	neg	neg	0	no	no	elbow
11	neg	neg	neg	1	multiple	yes	elbow
13	neg	neg	neg	0	hill	yes	diffuse
5	neg	neg	neg	0	no	yes	diffuse
4	neg	neg	neg	0	no	no	no
6	neg	neg	neg	0	no	yes	elbow hair
0	neg	nég	neg	NR	no	no	no
4	neg	neg	neg	0	hill	no	hair nail elbow
0	neg	neg	neg	0	gluteus	?	foot nail
5	neg	neg	neg	0	hill	yes	diffuse
0	neg	neg	neg	0	no	no	diffuse
6	neg	neg	40	1	hill	no	hair elbow
1	neg	neg	neg	1	no	yes	nail
6	neg	neg	neg	1	hill	no	hand
5	neg	neg	neg	0	no	no	diffuse
5	neg	neg	neg	0	no	no	diffuse
5	neg	neg	neg	0	wrist	yes	diffuse
5	nrg	neg	32	NR	no	yes	no
10	neg	neg	neg	0	hill	yes	hair
0	neg	neg	neg	1	no	yes	diffuse
1	neg	neg	neg	1	hill	no	elbow, foot
6	nrg	Pos22	neg	0	no	no	hair
10	neg	neg	neg	0	no	?	diffuse
0	neg	neg	neg	0	hill	?	elbow hair
4	neg	neg	neg	0	gluteus	yes	diffuse
5	neg	neg	pos 21	0	gluteus	yes	diffuse
4	neg	neg	neg	1	no	yes	knee
2	neg	neg	neg	1	no	?	diffuse
1	neg	neg	neg	0	no	yes	diffuse
6	neg	neg	34	1	no	no	hair
7	neg	neg	neg	0	no	yes	hand foot
0	neg	neg	neg	0	no	no	hand foot
27	neg	neg	34	0	no	yes	hair elbow
0	neg	neg	neg	NR	no	no	no
5	neg	neg	neg	0	no	no	diffuse
10	neg	neg	neg	0	no	yes	elbow nail
4	neg	neg	neg	0	no	no	hair
28	neg	neg	neg	1	hill	no	diffuse
9	neg	neg	neg	1	hill	yes	hair nail
3	neg	neg	32	0	no	no	hair
9	neg	neg	neg	NR	no	no	no
2	neg	neg	neg	0	no	no	hair
13	neg	neg	neg	0	no	no	diffuse
22	neg	neg	neg	0	0	no	foot
5	neg	neg	22	0	multiple	no	diffuse
0	neg	neg	neg	0	no	no	no
0	neg	neg	neg	1	no	no	diffuse
6	neg	neg	neg	0	multiple	no	elbow

12	neg	neg	neg	0	hill	no	diffuse
14	neg	neg	36	0	hill	yes	diffuse
0	neg	neg	neg	NR	no	no	no
3	neg	neg	neg	0	no	no	diffuse
2	neg	neg	neg	0	no	no	diffuse
0	neg	neg	neg	NR	no	no	no
5	neg	neg	neg	1	no	yes	diffuse
3	neg	neg	neg	0	hill	no	head
9	neg	neg	neg	0	no	yes	diffuse+++
5	neg	neg	neg	0	multiple	yes	diffuse
0	neg	neg	neg	1	no	yes	hair diffuse
4	neg	neg	neg	1	no	no	diffuse nail
0	neg	neg	neg	1	no	no	hair diffuse
8	neg	neg	neg	0	no	no	nail
20	neg	neg	neg	0	no	yes	diffuse
7	neg	neg	neg	0	elbow	no	hair diffuse
23	neg	neg	neg	0	no	yes	diffuse
0	neg	neg	neg	1	multiple	no	genital
31	neg	neg	neg	0	no	no	no
2	neg	neg	neg	0	no	no	nail
8	neg	neg	neg	1	no	no	hair hand
3	neg	neg	neg	1	hill	no	hair et elbow
2	neg	neg	neg	1	hill	yes	hair diffuse
5	neg	neg	neg	0	elbow	no	knee nail
4	neg	neg	neg	0	hill	no	hair knee elbow
0	neg	neg	22	1	no	no	hair diffuse
6	neg	neg	neg	0	no	no	diffuse
5	neg	neg	34	1	no	yes	hair diffuse
0	neg	neg	neg	0	no	no	no
0	neg	neg	neg	0	no	yes	hair
0	neg	neg	neg	1	hill	yes	no
10	neg	neg	neg	NR	no	no	no
3	neg	neg	neg	1	hill	yes	diffuse
6	neg	neg	neg	NR	no	no	no
7	neg	neg	neg	0	no	yes	diffuse
5	neg	neg	neg	0	no	no	diffuse

caspar			
Yes			
Yes			
Yes			
Yes			
Yes			
Yes			
Yes			
Yes			
Yes			
Yes			
Yes			
Yes			
Yes			
Yes			
Yes			
Yes			
Yes			
Yes			
Yes			
Yes			
Yes			
Yes			
Yes			
Yes			
Yes			
Yes			
Yes			
Yes			
Yes			
Yes			
Yes			
Yes			
Yes			
Yes			
Yes			
Yes			
Yes			
Yes			
Yes			
Yes			
Yes			
Yes			
Yes			
Yes			
Yes			
Yes			
Yes			
Yes			
Yes			

Yes			
Yes			
Yes			
Yes			
Yes			
Yes			
Yes			
Yes			
Yes			
Yes			
Yes			
Yes			
Yes			
Yes			
Yes			
Yes			
Yes			
Yes			
Yes			
Yes			
Yes			
Yes			
Yes			
Yes			
Yes			
Yes			
Yes			
Yes			
Yes			
Yes			
Yes			
Yes			
Yes			
Yes			
Yes			
Yes			
Yes			
Yes			
Yes			
Yes			
Yes			
Yes			
Yes			
Yes			
Yes			
Yes			
Yes			
Yes			
Yes			

Yes			
Yes			
Yes			
Yes			
Yes			
Yes			
Yes			
Yes			
Yes			
Yes			
Yes			
Yes			
Yes			
Yes			
Yes			
Yes			
Yes			
Yes			
Yes			
Yes			
Yes			
Yes			
Yes			
Yes			
Yes			
Yes			
Yes			
Yes			
Yes			
Yes			
Yes			
Yes			
Yes			
Yes			
Yes			
Yes			
Yes			
Yes			
Yes			
Yes			
Yes			
Yes			
Yes			
Yes			
Yes			
Yes			
Yes			
Yes			
Yes			

Yes			
Yes			
Yes			
Yes			
Yes			
Yes			
Yes			
Yes			
Yes			
Yes			
Yes			
Yes			
Yes			
Yes			
Yes			
Yes			
Yes			
Yes			
Yes			
Yes			
Yes			
Yes			
Yes			
Yes			
Yes			
Yes			
Yes			
Yes			
Yes			
Yes			
Yes			
Yes			
Yes			
Yes			
Yes			
Yes			
Yes			
Yes			
Yes			
Yes			
Yes			
Yes			
Yes			
Yes			
Yes			
Yes			
Yes			
Yes			
Yes			

Yes			
Yes			
Yes			
Yes			
Yes			
Yes			
Yes			
Yes			
Yes			
Yes			
Yes			
Yes			
Yes			
Yes			
Yes			
Yes			
Yes			
Yes			
Yes			
Yes			
Yes			
Yes			
Yes			
Yes			
Yes			
Yes			
Yes			
Yes			
Yes			
Yes			
Yes			
Yes			
Yes			
Yes			
Yes			
Yes			
